# Metastatic Colonization: Escaping Immune Surveillance

**DOI:** 10.3390/cancers12113385

**Published:** 2020-11-16

**Authors:** Julien Schaller, Judith Agudo

**Affiliations:** 1 Department of Cancer Immunology and Virology, Dana-Farber Cancer Institute, Boston, MA 02115, USA; Julien_Schaller@dfci.harvard.edu; 2 Department of Fundamental Oncology, University of Lausanne–Lausanne University Hospital, 1011 Lausanne, Switzerland; 3 Department of Immunology, Harvard Medical School, Boston, MA 02115, USA

**Keywords:** immune evasion, metastasis-initiating cell, cytotoxic T cell, myeloid cell, natural killer cell, dendritic cell, immune surveillance, disseminated tumor cell, anti-tumor immunity

## Abstract

**Simple Summary:**

Metastasis is the major cause of deaths in cancer. The determinants enabling metastatic colonization in various organs are an active field in tumor research. Notably, the immune system can eliminate tumor cells that disseminate to distant tissues but often fails, leading to metastasis. This review highlights known mechanisms involved in immune evasion by metastasis-initiating cells and gives insights in immune-metastatic cell interplay that has yet to be discovered.

**Abstract:**

Cancer immunotherapy has shifted the paradigm in cancer therapy by revitalizing immune responses against tumor cells. Specifically, in primary tumors cancer cells evolve in an immunosuppressive microenvironment, which protects them from immune attack. However, during tumor progression, some cancer cells leave the protective tumor mass, disseminating and seeding secondary organs. These initial disseminated tumor cells (DTCs) should potentially be susceptible to recognition by the immune system in the new host tissues. Although Natural Killer or T cells eliminate some of these DTCs, a fraction escape anti-tumor immunity and survive, thus giving rise to metastatic colonization. How DTCs interact with immune cells and the underpinnings that regulate imperfect immune responses during tumor dissemination remain poorly understood. Uncovering such mechanisms of immune evasion may contribute to the development of immunotherapy specifically targeting DTCs. Here we review current knowledge about systemic and site-specific immune-cancer crosstalk in the early steps of metastasis formation. Moreover, we highlight how conventional cancer therapies can shape the pre-metastatic niche enabling immune escape of newly arrived DTCs.

## 1. Introduction

Although novel therapies have emerged in recent years, metastasis remains the leading cause of death in cancer patients [1,2,3]. Radiotherapy and chemotherapy have been the standard of care in many cancers but have shown limited results in some patients. More recently, immunotherapies have emerged as a new pillar in cancer treatment, bringing promising results [4,5]. Nevertheless, these novel immune-based therapies only succeed in a fraction of patients. A better understanding of the crosstalk between the immune system and the metastatic cells, is needed to improve these therapies for metastatic cancer.

In 1889, Dr. Stephen Paget first described the “seed and soil” theory, in which tumor cells that spread become a “seed” that would favor some specific organ niches or “soil” in order to grow metastases [6]. Based on that concept, multiple subsequent studies investigated the features of the metastatic niche. In recent years, since the role of the immune system in cancer has been acknowledged, a new field is trying to characterize the immune-cancer cell crosstalk during metastasis [7,8]. Particularly, in order to grow into metastases, disseminated tumor cells (DTCs) must survive in diverse host organs outside the immunosuppressive environment of the primary tumor, hence evading immune surveillance. Thus, a fraction of cells that disseminate or DTCs may eventually give rise to metastases. These cells with such potential have been by some referred as metastatic stem or metastasis-initiating cells (MICs) [9]. The original concept of MICs was postulated as the metastatic outgrowth of specific human cancer cells isolated from primary tumor or blood, and reinjected into immunocompromised mice [10,11,12,13]. Unfortunately, these models did not enable to explore the roles of immunity against MICs, and so little is known about immune evasion during early stages of metastatic dissemination.

MICs evolve in a cellular environment different from the primary tumor and studies have characterized particular properties of such metastatic cells. In the tumor mass, cancer cells are likely under hypoxia and with limited nutrient supplies, whereas DTCs may not encounter those limitations [14]. Furthermore, cancer cells in the primary mass are surrounded by a different immune and stromal milieu than the one around MICs at metastatic sites [15,16]. Interestingly, some reports have shown that metastases can display a higher stemness profile compared to cancer cells in primary tumors, suggesting that MICs may co-opt immune escape abilities from stem cells [17,18]. Besides, tumor cells during dissemination undergo epithelial-to-mesenchymal transition [9,19] and it has been shown that mesenchymal phenotypes correlate with immunosuppressive tumors and reduce response to immunotherapy [20]. Thus, such diverging features of MICs and cancer cells in primary tumors likely result in molecular and cellular mechanisms providing MICs with immune evasion properties that are different from the escape mechanisms in a tumor mass.

Here we summarize the current knowledge on DTCs’ interactions with the immune system in the metastatic niche. In particular, we describe how primary tumors affect the function of the immune system at the organismal level, altering systemic immune responses, hence potentially supporting survival of DTCs outside the primary tumor microenvironment (TME). Furthermore, we focus on the recent studies exploring how MICs efficiently escape immunity, including cytotoxic T cell-mediated clearance. Finally, we discuss therapy-induced alterations of the pre-metastatic niche influencing metastatic spread, and novel immunotherapies potentially enabling to target DTCs.

## 2. Tumor-Mediated Systemic Immune Dysfunction

Development of novel immune-based therapies has fueled the interest in better understanding anti-cancer immunity. During tumor progression, gene mutations can lead to the production of modified proteins that can be recognized by the immune system, referred as neo-antigens. In order for such recognition to occur, these neo-antigens must be presented to T cells. In particular, dying cancer cells are engulfed by professional antigen-presenting cells, such as dendritic cells (DCs), which are most potent to mount an immune response. Upon antigen uptake, DCs travel to the tumor draining lymph nodes (LNs) where they encounter CD4+ and CD8+ T cells. Once there, DCs present antigens to and induce activation of T cells with a cognate T cell receptor (TCR). Primed T cells proliferate, leave the tumor draining LNs, circulate in the peripherical blood and finally reach the tumor mass. There, activated CD8+ T cells can recognize and kill tumor cells, while CD4+ T cells exhibit diverse functions that support an anti-tumor immune response [21,22,23,24,25,26].

Tumors can produce and secrete far-reaching signaling molecules including growth factors, and exosomes that alter the host systemically. Some of these factors result in changes that facilitate the survival and growth of cancer cells outside the primary tumor mass. Moreover, such molecules, mostly growth factors and cytokines, can re-shape the immune landscape at the organismal level [8,27,28,29]. For example, granulocyte colony-stimulating factor (G-CSF) and chemokines such as CCL2 and CXCL2 contribute to the mobilization of myeloid cell subsets from the bone marrow and to their recruitment to distant organs in cancer [8,29,30]. Notably, localized primary tumors can impact hematopoiesis, particularly myeloid cell formation such as granulocytes, monocytes and DCs. In particular, tumor-derived G-CSF leads to a systemic decline in DCs [31]. Furthermore, tumor-derived IL-6 and vascular endothelial growth factor (VEGF) are suggested to affect DC differentiation and maturation [32,33,34]. A notable tumor-mediated systemic effect on the immune system is impairment of DC function even in distant organs, although the exact mechanisms are yet to be uncovered. Thus, it has been shown in tumor-bearing animals that splenic DC activation is altered in response to bacterial infection. In these models, antigen presentation by DCs was reduced, and consequently, proliferation and cytotoxicity of CD8+ T cells in response to pathogens were dampened [35]. In agreement, circulating DCs exhibit lower antigen-presenting ability in breast cancer patients [36]. In addition to systemic elevation of cytokines that possibly impact DC functions, lipid accumulation in splenic DCs from cancer hosts may refrain the processing of antigens needed before the presentation to T cells [32,37]. Furthermore, beyond systemically abrogated DC function in the presence of a tumor, the number of circulating DCs is also decreased in breast and pancreatic cancer pre-clinical models due to alterations in DC development in bone marrow [31]. Interestingly, surgical resection of primary tumors was able to recover DC function and T cell activation [35]. Therefore, in cancer patients, altered antigen processing and presentation abilities of DCs may all contribute to an inefficient activation of T cells, thus possibly blunting the immune control of infections and tumor cells. Based on these data, tumor-associated dysfunction of DCs may weaken the immune surveillance of DTCs.

Moreover, beyond insufficient T cell activation due to reduced DC numbers or function, DCs play another key role orchestrating anti-tumor immunity. Notably, immature or alternatively activated DCs can induce antigen-specific tolerance via CD4+ T cell differentiation into regulatory T cells (Tregs) and/or induction of anergy in CD8+ T cells [38,39,40]. Tregs suppress anti-tumor immunity and can protect tumor cells from CD8+ T cell attack in the TME [41,42]. In cancer hosts, tumor-specific Tregs can circulate in peripherical blood [35,43], and may support cancer progression and dissemination to distant organs.

**Figure 1:**
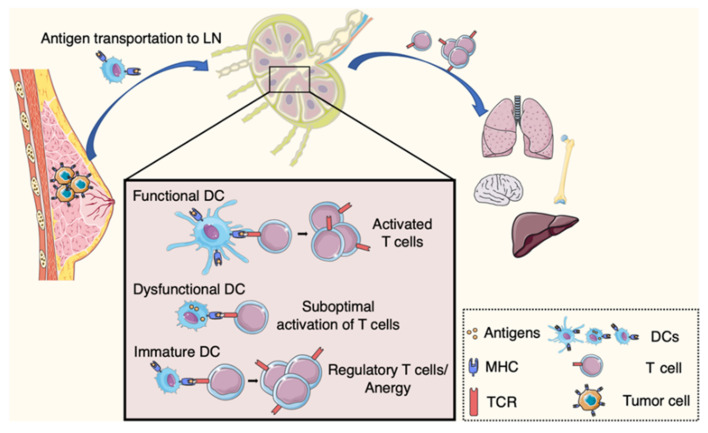
Adaptive immune response and systemic immune dysfunction in cancer. Upon engulfing tumor antigens dendritic cells (DCs) migrate to lymph nodes. There they initiate anti-tumor immunity, with the activation of CD8+ T cells. DC dysfunction in cancer hosts leads to inefficient T cell response. DCs can induce tolerance with the differentiation of CD4+ T cells into regulatory T cells. Altered systemic immunity affects immune niches in distant organs.

Together, these results suggest that the presence of a tumor can impact the systemic immune landscape (Figure 1), which influences the primary TME as well as the immune environment at pre-metastatic sites. Such systemic immunity dysfunction may then contribute to lessen immune clearance of cancer cells in diverse host organs, thus promoting metastasis establishment [35].

## 3. Immune Surveillance of Disseminated Tumor Cells (DTCs)

### 3.1. Influence of Host Organ in the Immune Landscape of Metastases

As previously discussed, tumors can exert systemic effects, modulating hematopoiesis and function of immune cells. A noticeable example is the effect of tumors on specific myeloid subsets that can modify the microenvironment in distant organs to make it more suitable for metastasis [8,35]. A large body of literature has characterized immune cells, mostly myeloid populations at pre-metastatic sites that foster tumor progression by providing recruitment or survival cues to DTCs [29,30,44,45,46,47]. Nonetheless, much less is known about the local anti-tumor immunity in the early metastatic environment after MIC colonization, and knowledge on how the pre-metastatic immune environment responds to the DTCs’ arrival is also incomplete. Indeed, tumor cells seeding secondary organs need to acquire several features that enable their survival, and metastatic outgrowth, including the ability to escape immune surveillance [7,9]. Epithelial tissues such as in lungs are frequently exposed to pathogens easily triggering immune responses, while organs such as brain or bones are less likely to encounter pathogens. Thus, the immune populations recruited in response to DTCs may greatly differ across organs. Interestingly, recent work showed that lung metastases have a higher immunogenic signature compared to metastases in bone, liver and brain [48]. Although a wide heterogeneity in metastatic immune environment, lung metastatic samples exhibited increased lymphocyte and DC infiltration consistent with the active local immune system in lungs [48]. In addition, immunogenic profiles in liver and brain metastases appeared to be independent of the primary tumor origin. This study suggests that the host organ of metastases has a notable effect on the local immune infiltrates [48]. Thus, immunotherapy guidelines may be based on metastatic immune signature rather than on the tumor of origin. Extrapolating these findings to the early metastasis process, we could speculate that site-specific cellular players in pre- and early metastatic niches shape the immune crosstalk with DTCs, hence favoring or lessening metastatic outgrowth. Therefore, cancer type is not the unique determinant of immune environment in metastases as pre-existing immune niches in distant organs may respond differently to metastatic colonization.

Abundance and quality of immune infiltrates in individual metastases have been correlated to progression of each individual lesion. Thus, it has been reported in a patient with ovarian cancer, that during chemotherapy treatment, regressing and stable metastases displayed high CD8+ and CD4+ T cell infiltration, as well as T cell clone expansion [49]. Yet, concomitant progressing metastatic lesions in the same individual showed T cell exclusion. Interestingly, CXCL9, chemokine contributing to T cell trafficking, was massively expressed in regressing and stable metastases [49]. This highlights the fact that response to therapy correlates to immune infiltration and this can differ across metastatic lesions in a single patient.

### 3.2. Myeloid Cell-Mediated Immunosuppression around DTCs

**Figure 2:**
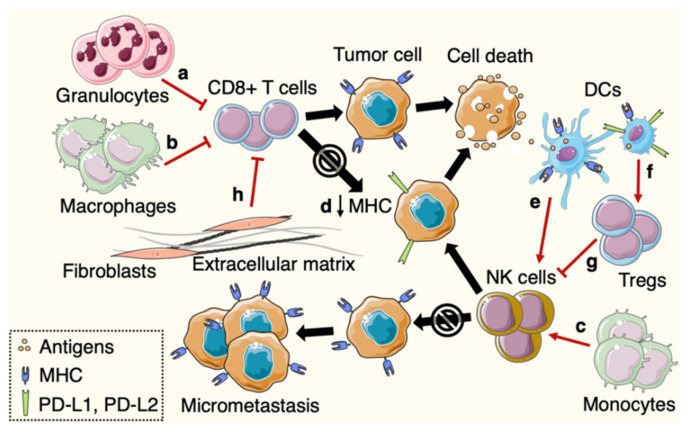
Intricate immune crosstalk in the metastatic niche and immune evasion by DTCs. Immune niche involving granulocytes and macrophages dampens CD8+ T cell function (**a**,**b**). DTCs that downregulate MHC class I can escape T cell-mediated surveillance (**d**). Immune control by NK cells is influenced by DCs, Tregs and monocytes (**c**,**e**–**g**). Fibroblasts may refrain T cell infiltration at the early metastatic site (**h**).

Immune surveillance by T cells may be dampened by myeloid populations that accumulate in the metastatic niche. In metastasis models, lung-recruited granulocytes directly inhibit cytotoxic T cells via inducible nitric oxide synthase (iNOS) expression, hence enhancing lung metastases (Figure 2a) [50]. Granulocyte recruitment in organs distant to the tumor mass is enabled by multiple cancer-immune crosstalk mechanisms [27,29,47,51]. Furthermore, p53 loss in cancer cells drives a systemic inflammatory cascade that recruits granulocytes to the lung pre-metastatic site. In particular, cancer-mediated WNT signaling stimulates IL-1β expression in tumor-associated macrophages (TAMs) leading to IL-17 production by activated ɣδ T cells, and consequently to systemic G-CSF increase [50,52]. Interestingly, G-CSF treatment in breast tumor animals results in DTC expansion and lung metastases [51]. Moreover, granulocytes can release arginase-1, leading to L-arginine degradation, which, in turns, may impact T cell function in the TME [53,54,55]. Thus, such mechanism may take place at the early metastatic sites that are enriched in granulocytes. Alternatively, tumors induce systemic increase in granulocytes and high granulocyte-to-lymphocyte ratio in peripherical blood is a marker of poor prognosis in many cancers [56,57,58]. Changes in granulocyte-to-lymphocyte ratio during immunotherapy may also predict patient outcome [59,60]. These observations imply that cancer cells drive systemic immune remodeling shaping the pre-metastatic environment, and increasing immune-suppressive granulocyte development. Thus, ensuing immune–immune crosstalk between primary tumors and metastatic sites impacts CD8+ T cell-mediated control of DTCs.

Macrophages also accumulate in the lung pre- and early metastatic niche. A distinct group of macrophages sustains metastatic cell survival in the lungs and can suppress cytotoxic CD8+ T cell function (Figure 2b) [61]. Conversely, a subtype of monocytes patrols in the lung vasculature and prevents metastatic seeding in melanoma and breast cancer models. In particular, the patrolling monocytes recruit and activate Natural Killer (NK) cells that have the innate ability to eliminate virus-infected and tumor cells. Thus, NK cells importantly contribute to immune surveillance of tumor cells (Figure 2c) [62]. Together, those data attest opposite roles of myeloid cell subsets in the immune control of DTCs. While most granulocytes and macrophages lessen immune control of metastasis and have been associated with DTC survival, other myeloid cell types may refrain metastatic spread by influencing NK cell immunity. Thus, the site-specific immune–immune crosstalk, itself influenced by cancer types and mutations, may affect the intrinsic susceptibility of each organ to metastasis formation.

### 3.3. Immune Evasion from Cytotoxic Immune Cells by Metastasis-Initiating Cells (MICs)

In murine melanoma and breast cancer models, CD8+ T cell anti-tumor immunity is suggested to successfully eliminate DTCs, since following depletion of T cells, metastasis outgrowth is promoted [51,63]. Thus, to grow into metastases, MICs must survive CD8+ T cell attack. To evade CD8+ T cell killing, tumor cells can upregulate the expression of programmed death-ligand 1 (PD-L1), known as a major immune checkpoint. During dissemination, tumor cells circulate in the blood and a fraction of them seed metastases. Elevated PD-L1 expression in such cells is associated with poor prognosis in patients with lung cancers, although studies remain controversial [64,65,66]. Similarly, expression of PD-L1 is increased in metastases compared to primary lesions in colorectal cancer [67]. Therefore, this suggests that DTCs expressing PD-L1 may resist CD8+ T cell-mediated elimination and may then initiate metastatic establishment. In pancreatic cancer, tumor cells disseminate to the liver and can stay in a latent state until permissive conditions enable metastasis outgrowth. Notably, DTCs reaching the liver are selected by adaptive immunity and most DTCs are eliminated. However, quiescent cancer cells lacking MHC class I escape T cell attack (Figure 2d) [68]. Indeed, MHC class I expression on tumor cells is necessary for antigen presentation and their recognition by CD8+ T cells. Thus, DTCs with insufficient MHC class I cannot be recognized and killed by CD8+ T cells. This mechanism of immune evasion by latent MICs mimics the mechanism previously described in quiescent epithelial stem cells [69], suggesting that MICs may co-opt immune evasive abilities from tissue stem cells. Furthermore, sufficient expression of other molecules in the antigen presentation pathway, including transporter associated with antigen processing (TAP) 1 and TAP2, is key for proper recognition and killing by T cells. Thus, downregulation of such proteins beyond MHC class I enables DTCs’ escape from T cell immunity. Several human cancer studies reported the association between downregulated expression of antigen presentation-mediating molecules and metastases. Moreover, the expression of antigen presentation proteins positively correlates with T cell infiltration in breast cancer [70,71,72]. While cancer cells are protected in the immunosuppressive TME, alterations in antigen presentation machinery may then be crucial for DTCs to escape CD8+ T cell attack in distant organs. However, tumor cells that lack MHC class I may be detected and killed by NK cells. Thus, a certain level of downregulation (but not complete loss) of MHC class I and other key proteins in the antigen presentation pathway may represent the most efficient cell-autonomous mechanism by MICs to evade both NK and T cell-mediated killing. In addition, other tumor mechanisms including actin cytoskeleton remodeling can alter immune-cancer cell interaction and participate in the immune evasion by MICs. In breast cancer cells, filamentous actin (F-actin) accumulates at the cell–cell junction between tumor and immune cells, so-called immune synapse. Such accumulation at the immune synapse has been linked with an increased resistance to Natural Killer (NK) cell lysis [73]. Interestingly, actin remodeling is increased in tumor cells undergoing epithelial-to-mesenchymal transition. Furthermore, autophagy contributes to elimination of cytotoxic molecule Granzyme B and could possibly contribute to decrease cancer cell susceptibility to NK cell-mediated killing [74]. Similar to expression changes of MHC class I and PD-L1 in cancer, modifications of cytoskeleton in DTCs, mimicking mechanisms involved in DCs, may contribute to their escape from cytotoxic immune cells [75,76].

### 3.4. Natural Killer (NK) Cell Function in Metastasis

NK cells serve as guardians against cancer and infected cells, and typically eliminate cells that lack MHC class I. In order to survive and build metastases, DTCs must escape NK cell surveillance. The NK cell killing ability is controlled by a fine-tuned signaling balance induced by NK activating receptors (including NKG2D) binding to their ligands on tumor cells, and by NK inhibitory receptors recognizing MHC class I. In cancer, expression of NKG2D ligands, such as UL16-binding proteins can be modulated at the transcriptional level, notably by IFNγ, DNA methylation, or low histone acetylation, and at the translational level, mostly through microRNAs [77,78]. Furthermore, NKG2D ligands can be cleaved from the cell surface by metalloproteinases or secreted in exosomes, thus reducing NK-cell mediated attack of cancer cells [79,80,81]. Malladi and colleagues described quiescent DTCs that downregulate ligands of NK activating receptors, thus evading NK cell attack in lung and breast cancers. Notably, those disseminated cells adopt stem cell features highly expressing Sox2 and Sox9 [82]. Moreover, Sox9 overexpression in metastatic lung tumor cells protects against NK killing by upregulation of MHC class I [18]. Interestingly, clusters of DTCs are less susceptible than single DTCs to NK cell-mediated clearance. Recent experimental evidence suggests that one possible mechanism for such resistance is that tumor cells forming clusters downregulate ligands of NK activating receptors [58]. Furthermore, clusters of tumor cells may recruit more immunosuppressive myeloid cells in the metastatic niche, which could also prevent NK cell attack [83,84]. Those studies highlight that DTC control by NK cells may depend on cancer cell features, such as proliferative state, and may then change throughout micro- and macrometastasis development.

### 3.5. DC–T Cell Crosstalk in the Early Metastatic Niche

Beyond downregulation of the antigen presentation machinery, DTCs can evade T cell immunity thanks to suppressive immune cells and signals that lessen T cell function. As mentioned above, altered DC functions in the early metastatic niche and draining LNs could affect T cell immunity and consequently, dampen immune clearance of DTCs [32,35,37]. Using a murine model of lung metastasis, Headley and colleagues demonstrated an initial response of myeloid cells after arrival of DTCs in the lungs. Multiple subsets of myeloid cells were recruited to the early metastatic site and were shown to engulf tumor-derived particles. Notably, in lung-draining LNs, tumor antigen-bearing cells mostly consisted of migratory conventional DCs [85]. Thus, DCs are the key myeloid cell type to carry tumor antigens to LNs to activate T cells. In particular, only a subset of conventional DCs referred as DC type 1 (DC1) is able to present antigens through MHC class I to CD8+ T cells in a process called cross-presentation. Thus, DC1 are essential for activation of cytotoxic CD8+ T cells [21,22]. Depletion of conventional DCs reveals enhanced metastatic colonization, further indicating DC roles in T cell effector function against DTCs [85]. Similarly, Batf3−/− mice which lack DC1 displayed increased metastasis in breast cancer. Besides T cell activation, DCs stimulate cytolytic activity of NK cells (Figure 2e). In particular, IL-12 produced by DCs stimulates IFNγ expression in NK cells and consequently, sustains NK cell-mediated control of metastasis [25,86]. Furthermore, upon sensing IFNγ, DCs secrete IL-12 that is required for success of immune checkpoint blockade therapy. Indeed, DC-derived IL-12 is critical for proper T cell activation and function [87]. Together, this illustrates that DC crosstalk with NK and T cells may build up immunity against DTCs. However, the ability of DCs to trigger an immune response may possibly be impaired by macrophages and granulocytes present at metastatic sites. For example, alveolar macrophages that accumulate in lungs during tumor progression decrease DC populations. Moreover, alveolar macrophages also reduce maturation of lung DCs, seen by downregulated expression of activating markers CD80 and CD86, in breast cancer models [88]. Targeting particular subset of myeloid cells in metastatic niches could then restore DC functions and possibly counteracts locally induced immune dysfunction that promotes lung metastasis.

Whereas DC-mediated T cell activation may be restrained by suppressive myeloid cells, a subpopulation of DCs expressing regulatory molecules, including PD-L1, lessens anti-tumor immunity. Those suppressive DCs are able to regulate T cell response in primary lung and colorectal cancers and potentially also reduce systemic immune surveillance of DTCs [89,90]. Similarly, a subset of DCs expresses high levels of ICOSL, PD-L1 and PD-L2 and is enriched in liver metastatic environment in pancreatic cancer. Those DCs induce immune tolerance via Treg development at the metastatic site (Figure 2f). Selective depletion of this suppressive DC subset rescues CD8+ T cell response and dampens metastasis [91]. Although DCs usually play a major role in T cell activation, some DCs abrogate the adaptive immune response and promote a tolerogenic environment. Depending on the anatomical site, the immune composition of the tumor-induced pre-metastatic niche possibly drives DC population towards an anti-tumor immunity suppressing or promoting phenotype.

### 3.6. Suppressive Lymphoid Cells in Metastasis

DC-mediated antigen presentation derives priming of CD4+ T cells and subsequent differentiation into T helper or, if tolerance is induced, into Tregs. Tregs remain less studied within the pre-metastatic niche, but recent studies have shown systemic induction of Tregs in cancer models. Notably, Tregs accumulate in lungs and LNs during tumor growth, whereas CD8+ T cell population fluctuates in LNs [35,92]. Therefore, such altered LN composition in T cell subsets may impair the initiation of anti-tumor immunity and sustain metastasis progression. This is supported by the fact that accumulation of Foxp3+ Tregs in draining LNs is associated with LN micrometastases and shorter relapse-free survival in patients with breast cancer [93]. Similarly, elevated Treg density in draining LNs correlates with LN metastases in gastric and colorectal cancers, suggesting that Tregs could collaborate to an immunosuppressive environment in pre-metastatic LN niche [94,95]. Interestingly, a study revealed a tumor-induced subset of “regulatory B cells” able to favor differentiation of resting CD4+ T cells into Tregs. By means of Treg suppressive functions, regulatory B cells then promote metastasis formation in breast cancer [92,96]. In addition to their role in the suppression of effector T cells, Tregs are suggested to eliminate NK cells, hence enabling lung metastases in breast cancer. Indeed, in this setting, NK cells correctly prevented lung metastasis progression in T cell deficient mice. These studies indicate that Treg–NK cell crosstalk in distant tissues interferes with immune control of DTCs (Figure 2g) [86,92,97].

Besides the systemic suppression by tumor-specific Tregs, the differentiation of naïve CD4+ T cells into T helper (Th) 1 or Th2, driving inflammatory and anti-inflammatory responses, respectively, influences cancer progression. In particular, a disbalance between Th1 and Th2 differentiation may lessen immune surveillance in the lungs [98]. For example, oxygen sensing proteins expressed in T cells dampen Th1 immune response in lungs and are critical to maintain an immunoregulatory state that prevents unnecessary inflammation in response to innocuous foreign antigens. However, repression of Th1 in favor of Th2 immunity induces an immune-permissive environment that fosters metastatic colonization [98]. Similarly, the previously described myeloid cell populations in the pre-metastatic niche also foster T cell differentiation into anti-inflammatory Th2 cells [98,99]. Together, there is evidence that multiple lymphoid cell populations participate in the establishment of a permissive milieu that suppresses NK and T cell-mediated immune surveillance, thus supporting metastasis expansion.

### 3.7. Immune–Stromal Crosstalk in Metastasis

Cancer cell features including the ability to interplay with stromal components promote metastasis. For example, upon arrival of DTCs at secondary sites, cancer–stroma crosstalk induces stromal periostin expression, supporting metastatic colonization [100]. In lungs of breast cancer models, fibroblasts exhibit an inflammatory phenotype and secrete chemokines that sustain metastasis [101]. Similarly, fibroblasts in primary breast cancers secrete chemokines promoting tumor migration and invasion [102,103]. Besides tumor–stroma interactions, activated cancer-associated fibroblasts (CAFs) influence anti-tumor immune response in the TME and restrict T cell infiltration into the tumor through extracellular matrix deposition [104]. In the TME, increased transforming growth factor (TGF) β drives T cell exclusion and inhibition of TGFβ potentiates immunotherapy as well as prevents metastasis in colorectal cancer models [105]. Extrapolating these findings to the early metastatic niche, we could speculate that fibroblasts limit T cell infiltration in diverse organs following metastatic seeding. However, specific stromal cell influences on T cell-mediated surveillance of DTCs remain to be explored (Figure 2h).

## 4. Therapy-Associated Pre-Metastatic Niche Modifications and Future Therapeutic Targets

How commonly used chemotherapy regimens affect pre-metastatic niche and metastasis formation has become an active field in cancer research. Chemotherapy fails to prevent tumor progression and recurrence in many cancers and recent evidence suggests it may support metastasis [106]. In breast cancer models, paclitaxel chemotherapy promotes lung metastases. Interestingly, in tumor-bearing mice, paclitaxel treatment leads to chemokine-mediated pulmonary recruitment of monocytes which trigger an undesired pro-metastatic side-effect. Moreover, chemotherapy also reduces expression of perforin in NK and T cells, thus affecting their cytotoxic function in lungs [107,108,109]. Together, this highlights substantial impact of chemotherapy on immune components in pre- and metastatic tissues. Therefore, chemotherapy may foster further accumulation of immunosuppressive myeloid populations at pre-metastatic sites and may concomitantly contribute to the reprograming of immune cells within distant organs, hence lessening immune surveillance of DTCs. However, further studies are needed to elucidate the different effects of each chemotherapy agent and of combination therapies on the immune system.

Alternatively, therapies targeting the pre-metastatic niche may help restoring immune control of MICs. In models of pulmonary metastases, DNA methyltransferase and histone deacetylase inhibitors following primary tumor resection modify the pre-metastatic niche [110]. By downregulating CCR2 and CXCR2 such adjuvant epigenetic therapies dampen lung accumulation of monocytes and granulocytes that are able to suppress T cell activity. Thus, this results in longer survival and reduced metastasis [110]. Furthermore, immunotherapy could be specifically directed towards MICs in order to prevent or eliminate metastases. Different therapy approaches targeting primary tumor-initiating cells, including MUC1 and MESO CAR-T cells, are under clinical trials [111]. The future development of immunotherapies may then improve late stage cancer treatment by disrupting the immunosuppressive metastatic niche or directly by increasing T cell response directed against MICs.

## 5. Conclusions and Perspectives

The above-mentioned studies indicate how the immune system responds to the presence of DTCs in distant tissues and how the tumor-associated changes in systemic and site-specific immunity influence metastasis. Besides impacting the systemic immune response, primary tumor growth induces formation of immune pre-metastatic niches supporting the seeding and survival of DTCs.

Although both NK and cytotoxic T cells certainly succeed in eliminating some DTCs, alterations of their function by suppressive macrophages and granulocytes at early metastatic sites may contribute to failure in the clearance of DTCs, hence promoting later development of metastases (Figure 2). The accumulation of other suppressive immune cells, including Tregs, in pre-metastatic niches may also lessen CD8+ T and NK cell abilities to kill DTCs. As immune environments in overt metastases substantially differ between organs, it is likely that immune pre- and early metastatic niches also depend on the host organs. Thus, although mostly studied in lungs, mechanisms involved in abrogating immune surveillance of DTCs may involve different immune and stromal cells according to the location of metastases. How site-specific immune populations evolve through tumor progression and affect immune escape by MICs still needs further investigations. Interestingly, latent DTCs also exhibit distinctive features to escape cytotoxic T cell recognition or NK cell attack. However, cell-autonomous mechanisms used by MICs remain to be fully explained. A greater understanding of hallmarks of MICs evading innate and adaptive immunity could then open future therapeutic opportunities to specifically target tumor cells capable of initiating metastasis.
